# Pharmaceutical intervention in the rational use of intravenous omeprazole

**DOI:** 10.31744/einstein_journal/2020AO4433

**Published:** 2019-12-20

**Authors:** Eduardo Silva Araújo, Ana Carolina Figueiredo Modesto, Tatyana Xavier Almeida Matteucci Ferreira, Mércia Pandolfo Provin, Dione Marçal Lima, Rita Goreti Amaral

**Affiliations:** 1 Faculdade de Medicina, Universidade Federal de Goiás, Goiânia, GO, Brazil; 2 Hospital das Clínicas de Goiás, Universidade Federal de Goiás, Goiânia, GO, Brazil; 3 Faculdade de Farmácia, Universidade Federal de Goiás, Goiânia, GO, Brazil

**Keywords:** Omeprazole, Drug utilization, Pharmaceutical services, Infusions, intravenous, Patient safety

## Abstract

**Objective:**

To describe the pharmaceutical interventions of a vertical clinical pharmacy service to promote the rational use of intravenous omeprazole.

**Methods:**

A prospective and descriptive study carried out at a university hospital in the Midwestern Region of Brazil, from November 2014 to May 2015. The service consisted of the analysis of adequacy of the route of administration of omeprazole in relation to the clinical conditions of the patient, as well as the use of the appropriate diluent. Interventions were recorded in medical records and subsequently evaluated for acceptance.

**Results:**

A total of 770 prescriptions were evaluated. Interventions related to diluent replacement were more accepted (p<0.001), and surgeons were the specialty that used the intravenous route inappropriately (p<0.001).

**Conclusion:**

Although partially accepted, pharmaceutical interventions could contribute to improve patient safety, since they allowed the use of a safer route of administration.

## INTRODUCTION

The inappropriate use of medications can lead to the occurrence of adverse drug event (ADE), raising the morbidity and mortality rates, besides increasing the costs of care for healthcare systems.^(^^1^^)^

The hospital environment is more susceptible to ADE due to the quantity and variety of medications used. Studies have shown that 38% of adverse events that occur within this environment are drug-related.^(^^2^^,^^3^^)^ In a hospital environment, the intravenous route (IV) is a great source of ADE, since it is commonly used for prescriptions to inpatients.^(^^4^^)^

The choice of the parenteral route with no precise or justified indication represents an obstacle to the rational use of medications. This route shows potential risks, such as infection, impossibility of reversal because of the immediate pharmacological effect, propensity towards intoxication and possibility of thromboembolic events.^(^^5^^,^^6^^)^ The need for dilution is also a source of error. There are reports of incorrect use of diluents in IV preparations, such as inadvertent use of concentrated electrolyte solutions to reconstitute medications, which is a serious error with the potential to lead to death.^(^^7^^)^

In addition to the risks, the IV route cost is up to five times higher than oral administration, besides indirect costs, such as diluents, equipment for administration, and a longer time of implementation in the work of the nursing team.^(^^8^^,^^9^^)^

Added to these factors, it is known that the proton pump inhibitors (PPI) are the class of medications most prescribed all over the world,^(^^10^^)^ and its prolonged use can lead to several undesired effects, such as pneumonia, infections by *Clostridium difficile*, osteoporosis, and fractures in the elderly, besides being responsible for many drug interactions.^(^^11^^)^ Some studies showed that more than 50% of indications for PPI, both by oral route (PO) and by IV route at hospitals are inappropriate.^(^^12^^,^^13^^)^

There is no evidence as to the superiority of PPI administered PO in comparison with the IV route. Additionally, their PO administration is even more cost-effective, since it contributed to a decrease in the hospital inpatient stay.^(^^14^^)^ Considering the administration of medications via PO as a safer practice, sequential therapy (ST), which consists of switching from the IV formulation to the PO as soon as the patient presents with clinical conditions for such,^(^^8^^)^ can be a strategy to be adopted in healthcare organizations.

Bearing in mind that the presence of a clinical pharmacist in the multidisciplinary teams is a safety strategy that institutions have adopted for the prevention of ADE,^(^^15^^)^ the promotion of the correct use of the IV route of PPI can contribute towards the decrease in morbidity and mortality related to the use of medications in the hospital environment.

## OBJECTIVE

To describe pharmaceutical interventions in a vertical clinical pharmaceutical service for the promotion of the rational use of omeprazole using the intravenous route.

## METHODS

This is a prospective and descriptive study conducted at a clinical pharmacy service of a university hospital in the Midwestern Region of Brazil. The data collection period was between November 2014 and May 2015. The project was approved by the Research Ethics Committee (CEP) of the organization, under opinion no. 810.341 and CAAE: 35951214.0.0000.5078. The present study was exempted of application of the Informed Consent Form (ICF) by CEP. The vertical pharmaceutical service, object of this study, consisted of the systematic evaluation of intravenous omeprazole in clinical and surgical medicine at the organization. Omeprazole was the PPI of choice, since it is the only representative of this class of drugs available with the standardization of the organization. Such clinics were chosen to provide the service since they are hospitalization units that receive patients from several specialties, and their hospital stay is long. Both inpatient units have 60 beds each.

Patients included in the study were those hospitalized in the clinical or surgical units during the study period, and whose prescriptions contained omeprazole IV; excluded were those whose patient records were not found. The medical records were indispensable for evaluating if the patient's clinical condition was in accordance with the use of the IV route. At the study organization, no electronic medical record was used, and every assessment was done by means of physical medical records.

Daily, in the pharmacy, the pharmacist checked the prescriptions containing intravenous omeprazole from the inpatients units selected for the study. With these prescriptions, he/she would go to the inpatient unit and search for the patient's medical records.

With the medical records in hands, the pharmacist's first activity was to evaluate the prescription of the omeprazole diluent, whether the diluent used was the correct one to utilize in the reconstitution, provided together with the product. An “inappropriate diluent” was considered when on the prescription any other diluent was written in addition to that which was appropriate for the product. In the case of non-conformity with the diluent prescribed, the pharmacist would document in the medical record, instructing the prescriber about the correct diluent for drug reconstitution.

The second activity carried out consisted of evaluating the appropriateness of omeprazole administration route. For the purposes of this study, an “inappropriate administration route” was considered when the intravenous omeprazole was prescribed to patients who met the following requirements: being afebrile for 24 hours; not on vasopressors; not presenting with problems in gastrointestinal absorption and gastrointestinal mobility; having a prescription for oral diet and tolerating the food; not on antiemetics over the last 24 hours; and be using other PO medications.^(^^9^^,^^16^^)^ For such patients, the pharmacists would make a note in the medical record to the prescriber instructing to substitute IV for PO administration, since this is more adequate for the patient's clinical status.

After the evaluations and the respective annotations in the medical records, the pharmacist monitored the patient's prescriptions to verify compliance of the prescriber with the recommendations for a period of 7 days. The attitude of the prescriber after instructions of the pharmacist was categorized as “intervention accepted,” when the prescriber modified the route of administration or the diluent as per orientations from the pharmacist, or “intervention not accepted” when, after the pharmacist's instructions, the prescriber maintained the route of administration or the diluent. When there was no compliance, the pharmacist would make a new prescription evaluation after this period and record it in the medical records.

Data were collected by means of search in medical records and specific forms developed by the Vertical Clinical Pharmacist Service. The information generated was processed and analyzed by means of the software EpiInfo™ version 3.5.4, and STATA version 12.0. Associations among the variables were tested using Pearson's χ^2^ test, Fisher's exact test, and McNemar's test with a significance level of 5%.

## RESULTS

A total of 978 patient prescriptions were included during the study period containing intravenous omeprazole. Of these, 10 were excluded because the patients had been discharged, and 198, due to unavailability of the patient's medical records at the time of the pharmacist's evaluation, totaling up 770 analyzed prescriptions.

Prescriptions of 337 patients were analyzed. A slight predominance of female patients was noted (Table 1). The mean age of patients was 55.12 years (±18.01 years), more than half the patients were under 60 years of age, and increase in age proved to be a protective factor (p=0.038) for the inadequate IV route use. As to the specialty of the prescriber, the inappropriateness of omeprazole administration route predominated among those with surgical specialties.

**Table 1:** Prevalence of inappropriate administration route and diluent for intravenous omeprazole

Variáveis		n=337	Inappropriate route			Inappropriate diluent		
		n (%)	n (%)	OR (95%CI)	p value	n (%)	OR (95%CI)	p value
Sex								
	Male	153 (45.40)	40 (26.14)	0.92 (0.82-1.04)	0.189*	12 (7.84)	1.00	0.432*
	Female	184 (54.60)	37 (20.11)	1.00		19 (10.33)	0.97 (0.90-1.04)	
Age, years								
	Up to 39	77 (22.85)	12 (15.58)	1.00	0.038*	7 (9.09)	0.99 (0.90-1.09)	0.955*
	40-59	105 (31.16)	20 (19.05)	0.95 (0.83-1.09)		9 (8.57)	1.00	
	Over 60	155 (45.99)	45 (29.03)	0.84 (0.73-0.96)		15 (9.68)	0.98 (0.91-1.06)	
Specialty of the prescriber								
	Medical	118 (35.01)	40 (33.90)	1.00	0.000*	13 (11.02)	0.96 (0.89-1.04)	0.397*
	Surgical	219 (64.99)	37 (16.89)	1.25 (1.09-1.44)		18 (8.22)	1.00	

* Pearson's χ^2^ test.

OR: odds ratio; 95%CI: 95% confidence interval.

In 13.12% (n=101) of prescriptions evaluated, the administration route for omeprazole was considered inadequate for the patient's clinical condition. Among the prescriptions with administration routes considered adequate, the restriction of oral diet (514; 73.22%), motility problems (143; 20.37%), and absorption problems (45; 6.41%) were the most frequent reasons. It is noteworthy that in some cases, the patients presented with more than one reason to justify the adequacy of IV use.

As to the adequacy of the prescribed diluent, in 5.06% (n=39) of prescriptions evaluated, inappropriate type of diluent was noted. We pointed out that in 100.00% (n=39) of cases of inadequacy of the type of diluent, we observed that it was with distilled water.

Of a total number of 101 prescriptions assessed by the pharmacist, who considered the IV administration route as inadequate, in 39.60% (n=40) of cases, the intervention was accepted by the prescriber. As to 29 prescriptions in which the pharmacist verified the use of an inadequate diluent, 44.83% (n=13) of interventions were accepted by the prescriber. The pharmaceutical intervention for substitution of medication dilutent was better accepted by prescribers than the intervention for adequacy of the administration route (p=0.000) (Table 2).

**Table 2:** Prevalence of acceptance of pharmaceutical interventions as to sequential therapy and substitution of intravenous omeprazole diluent

Interventions	Sequential therapy	Diluent substitution	p value
Accepted	40 (39.60)	13 (44.83)	0.000*
Not accepted	61 (60.40)	16 (55.17)	
Total	101	29	

Results expressed as n (%).

* McNemar test.

## DISCUSSION

The results of this study demonstrated that pharmaceutical interventions could collaborate for the correct use of drugs in a hospital environment. Other studies that proposed monitoring the adequacy of medication administration route by the pharmacist also noted the importance of ongoing accompaniment in the promotion of rational use of IV drugs.^(^^17^^)^ It is known that the presence of the pharmacist is a safety strategy,^(^^15^^)^ since it contributes to promoting the correct use of medications in the hospital setting.^(^^18^^)^

Although the presence of the pharmacist is, in fact, a strongly recommended and accepted safety strategy in other countries, this is not true for a large part of Brazil. The pharmacist faces difficulties both in support by managers, and in acceptance on the part of their own multiprofessional team.^(^^19^^)^ This reality was also observed in the present study, since the pharmaceutical interventions more related to the clinical evaluation made by the pharmacist, had lower compliance on the part of the team.

In general, the acceptance of pharmaceutical interventions in this study, when compared to those of another Brazilian organization,^(^^20^^)^ were discrepant, since in this study the authors observed a lower intervention acceptance rate. Such findings may be partially attributed to the fact of communication of the pharmaceutical interventions being documented in the medical records, and for the time interval determined for reevaluation by the pharmacist. These factors may have contributed towards the lack of compliance with the interventions. Additionally, the difference in recognizing the role of pharmacists in both organizations, and the potential of this type of service contributing to safety of the medication process and prevention of ADE^(^^15^^)^ may have also contributed towards these results.

The low compliance with interventions in reference to appropriateness of administration route can also be due to the fact the IV route is preferentially used at hospitals. This is a route in which the medication already presents with an immediate therapeutic response, it is convenient for the patient, already having been punctured, and therefore, with a quick access available.^(^^6^^)^ It is noteworthy that despite the great practicality, inappropriate use of intravenous route can lead to other problems that impact the quality of care, such as phlebitis and bloodstream infection.^(^^8^^)^

Moreover, the errors resulting from the medication process also contribute towards an increase in morbidity and mortality of inpatients. The reconstitution of antimicrobials with potassium chloride has been used in Brazilian hospitals^(^^7^^)^ and, although rare, the outcomes are mostly fatal. Safe practices for the use of the IV route should be adopted, encouraged, and widely propagated in healthcare organizations. Such an administration route, in addition to the risks presented, is costlier, as it involves direct costs with diluents, syringes, needles, intravenous infusion devices, besides the indirect costs with time of the nursing team that prepare the medications.^(^^8^^,^^9^^)^

On the other hand, the acceptance of pharmaceutical interventions in reference to pharmacotechnical aspects of therapy, as well as the acknowledgement of the mistake related to the diluent, can be better understood by part of the team. This fact suggests, for these teams, the role of the pharmacist is more closely related to the formulation of the medications than to the clinical practice, since the syllabus components of the undergraduate Pharmacy course represents a barrier to one's clinical practice, from the medical point of view.^(^^21^^)^

Even though the evaluation of the diluent does not demand essentially clinical knowledge on the part of the pharmacist, in the case of omeprazole, repercussions of the use of the inadequate diluent can influence the health outcomes. It is known that stability of omeprazole is pH-dependent, and its reconstitution should only be done with the proper diluent that accompanies the product. When any other diluent is used in the reconstitution of omeprazole, it may interfere in stability of the final solution, and efficacy of the medication can be hindered.^(^^22^^)^

The different characteristics of the professionals of medical and surgical specialties have proved to be evident in this study. The adequate choice of administration route of the medication presupposes a closer doctor-patient relationship, which is commonly observed in medical specialties.^(^^23^^)^ On the other hand, the marked characteristic of surgeons, which is practicality and pragmatism,^(^^24^^)^ may not favor teamwork. It is necessary to promote strategies to make the members of this specialty more receptive to the strategies related to safe use of medications.

As to age as a protective factor for the inadequate use of the IV route, such finding may be associated with the fact that usually the medical team has greater care when prescribing for elderly patients.^(^^25^^)^ Additionally, whenever possible, the IV route should be avoided in elderly patients. However, when there is a need to administer drugs by the parenteral route, it is important to evaluate the subcutaneous route - a technique commonly known as hypodermoclysis, an alternative to the IV route.^(^^26^^)^ These findings can be further explained by results of the study, since medical specialties were closely associated with care of elderly patients at the hospital of the study.

The weaknesses of this study comprise the organization not having electronic medical records, which may have contributed towards the low level of compliance with the pharmaceutical interventions, since these were recorded in the physical medical records and were only read by part of the medical team on their next visit. Another limitation also observed was the workload of the pharmacists to fulfill their activities, *i.e*., 10 hours a week. It is important to point out that, even without available technological resources and a limited workload, the pharmacist was capable of evaluating an expressive number of prescriptions and making the respective annotations in the medical record, besides recording the interventions. This model of service can serve as subsidies for organizations that do not count on electronic records to implement a clinical pharmacy service.

As strengths, we point out the prospective nature of the evaluation, which eliminates the characteristic biases of retrospective investigations, in addition to the fact that the vertical clinical pharmacist service was implemented in two inpatients units of a single organization. Perspectives of future studies on this theme could, in addition to evaluating the adequacy of the PPI administration route, check the indications for use, for there are few studies that have evaluated the rational use of this class of medications in hospital environments.

Training and sensitization programs of the teams as to the rational use of PPI should be implemented. Although these medications are widely used in clinical practice, a strict evaluation of the risk-benefit could be a strategy for preventing future complications related to their prolonged use.

Within this context, the pharmacist could contribute with the team in several ways: collaborating in the preparation of protocols and guidelines for use of medications at the organization, in sensitization and training as to the rational use of the medications, and participating in the visits or in the dispensation of the medications.

## CONCLUSION

The pharmaceutical interventions for promoting the rational use of intravenous omeprazole, although partially accepted, were able to contribute with the improvement of patient safety, since they allowed the use of a safer administration route. There were differences in acceptability of the pharmaceutical interventions related to the use of the correct diluent in detriment of those related to the exchange of the administration route. Inadequate use of the intravenous route was greater among surgeons in comparison to clinicians, and increased age proved to be a protective factor for the correct use of the intravenous route.
