# Supplementary material for: Pharmaceutical intervention in the rational use of intravenous omeprazole
Source: Einstein (Sao Paulo). 2019 Dec 20;18:eAO4433. doi: 10.31744/einstein_journal/2020AO4433 (PMC6924820; doi:10.31744/einstein_journal/2020AO4433)
Supplement: Translation

# Translation: Intervenção farmacêutica no uso racional de omeprazol intravenoso

**Authors:** Eduardo Silva Araújo, Ana Carolina Figueiredo Modesto, Tatyana Xavier Almeida Matteucci Ferreira, Mércia Pandolfo Provin, Dione Marçal Lima, Rita Goreti Amaral

**Affiliations:** 1 Faculdade de Medicina, Universidade Federal de Goiás, Goiânia, GO, Brasil; 2 Hospital das Clínicas de Goiás, Universidade Federal de Goiás, Goiânia, GO, Brasil; 3 Faculdade de Farmácia, Universidade Federal de Goiás, Goiânia, GO, Brasil

**Keywords:** Omeprazol, Uso de medicamentos, Assistência farmacêutica, Infusões intravenosas, Segurança do paciente

## Abstract

**Objetivo:**

Descrever as intervenções farmacêuticas de um serviço farmacêutico clínico vertical, para a promoção do uso racional do omeprazol intravenoso.

**Métodos:**

Estudo prospectivo e descritivo realizado em um hospital universitário da região Centro-Oeste do Brasil, no período de novembro de 2014 a maio de 2015. O serviço consistia na análise da adequabilidade da via de administração do omeprazol em relação às condições clínicas do paciente, bem como a utilização do diluente adequado. As intervenções eram registradas em prontuário e, posteriormente, avaliadas quanto à aceitação.

**Resultados:**

Foram avaliadas 770 prescrições. As intervenções relacionadas à substituição do diluente foram mais aceitas (p<0,001), e os cirurgiões foram a especialidade que utilizou a via intravenosa de maneira inadequada (p<0,001).

**Conclusão:**

Embora parcialmente aceitas, as intervenções farmacêuticas puderam contribuir com a melhoria da segurança dos pacientes, uma vez que permitiram a utilização de uma via de administração mais segura.

## INTRODUÇÃO

O uso inapropriado de medicamentos pode levar à ocorrência de eventos adversos a medicamentos (EAM), elevando as taxas de morbidade e mortalidade, além de aumentar os custos da assistência à saúde para os sistemas de saúde.^(^^1^^)^

O ambiente hospitalar é mais suscetível aos EAM pela quantidade e pela variedade de medicamentos utilizados. Estudos mostram que 38% dos eventos adversos que ocorrem nesse ambiente estão relacionados aos medicamentos.^(^^2^^,^^3^^)^ Em ambiente hospitalar, a via intravenosa (IV) é uma grande fonte de EAM, por ser comumente empregada entre as prescrições destinadas a pacientes internados.^(^^4^^)^

A escolha da via parenteral sem indicação precisa ou justificada representa obstáculo ao uso racional de medicamentos. Essa via apresenta riscos potenciais, como infecção, impossibilidade de reversão devido ao efeito farmacológico imediato, facilidade de intoxicação e possibilidade de eventos tromboembólicos.^(^^5^^,^^6^^)^ A necessidade de diluição também é uma fonte de erro. Existem relatos da utilização de diluentes de forma errônea em preparações IV, como no caso do uso inadvertido de soluções concentradas de eletrólitos para reconstituição de medicamentos, constituindo erro grave, com potencial de provocar óbito.^(^^7^^)^

Além dos riscos, a via EV apresenta também um custo até cinco vezes maior que a oral, possuindo também custos indiretos, como diluentes, equipamentos para administração e um tempo maior de operacionalização no trabalho da equipe de enfermagem.^(^^8^^,^^9^^)^

Somado a esses fatores, sabe-se que os inibidores da bomba de prótons (IBP) são a classe de medicamentos mais prescrita no mundo,^(^^10^^)^ e seu uso prolongado pode acarretar diversos efeitos indesejados, como pneumonia, infeções por *Clostridium difficile*, osteoporose e fratura em idosos, além de ser responsável por diversas interações medicamentosas.^(^^11^^)^ Em ambiente hospitalar, estudos têm mostrado que mais de 50% das indicações de IBP, tanto por via oral (VO) quanto por IV, são inapropriadas.^(^^12^^,^^13^^)^

Não existem evidências quanto à superioridade dos IBP administrados por VO em comparação à via IV. Além disso, sua administração por VO mostrou-se ainda mais custo-efetiva, uma vez que contribuiu para a diminuição dos dias de internação dos pacientes.^(^^14^^)^ Considerando a administração de medicamentos por VO uma prática mais segura, a terapia sequencial (TS), que consiste na troca da apresentação IV pela VO assim que o paciente apresenta condições clínicas para tal,^(^^8^^)^ pode ser uma estratégia a ser adotada nas instituições de saúde.

Considerando que a presença do farmacêutico clínico nas equipes multidisciplinares consiste em estratégia de segurança que as instituições de saúde têm adotado para a prevenção de EAM,^(^^15^^)^ a promoção do uso correto da via IV dos IBP pode contribuir para a diminuição da morbimortalidade relacionada ao uso dos medicamentos em ambiente hospitalar.

## OBJETIVO

Descrever as intervenções farmacêuticas de um serviço farmacêutico clínico vertical para a promoção do uso racional do omeprazol por via intravenosa.

## MÉTODOS

Trata-se de estudo prospectivo e descritivo conduzido em um serviço de farmácia clínica em um hospital universitário da Região Centro-Oeste do Brasil. O período de coleta de dados foi entre novembro de 2014 a maio de 2015. O projeto foi aprovado pelo Comitê de Ética em Pesquisa (CEP) da instituição, sob parecer 810.341 e CAAE 35951214.0.0000.5078. O presente estudo foi isento da aplicação do Termo de Consentimento Livre e Esclarecido (TCLE) por parte do CEP. O serviço farmacêutico clínico vertical, objeto deste estudo, consistia na avaliação sistemática do omeprazol intravenoso na clínica médica e cirúrgica na instituição. O omeprazol foi o IBP de escolha por ser o único representante dessa classe de medicamentos disponível na padronização da instituição. Tais clínicas foram escolhidas para a oferta do serviço por se tratarem de unidades de internação que recebem pacientes de diversas especialidades, e o período de permanência deles ser prolongado. Ambas as unidades de internação possuem 60 leitos cada uma.

Os pacientes incluídos no estudo foram àqueles internados na clínica médica ou cirúrgica no período do estudo, cujas prescrições continham o omeprazol IV; foram excluídos aqueles cujo prontuário do paciente não foi localizado. O prontuário era indispensável para avaliar se as condições clínicas do paciente estavam em acordo com o uso da via IV. Na instituição em estudo, não se utilizava prontuário eletrônico, e toda a avaliação foi feita por meio de prontuários físicos.

Diariamente, o farmacêutico verificava, na farmácia, as prescrições que continham omeprazol intravenoso das unidades de internação selecionadas para o estudo. De posse dessas prescrições, dirigia-se para a unidade de internação, em busca do prontuário do paciente.

De posse do prontuário, a primeira atividade do farmacêutico consistia na avaliação da adequação da prescrição do diluente do omeprazol, quando era avaliado se o diluente utilizado para sua reconstituição era o diluente próprio, fornecido com o produto. Foi considerado “diluente inapropriado” se, na prescrição, constasse qualquer outro diluente, além daquele próprio para o produto. Caso encontrasse alguma não conformidade quanto ao diluente prescrito, o farmacêutico realizava anotação no prontuário, orientando ao prescritor o uso do diluente correto para reconstituição do medicamento.

A segunda atividade realizada consistia na avaliação da adequação da via de administração do omeprazol. Para fins deste estudo, foi considerada como “via de administração inapropriada” quando o omeprazol intravenoso estava prescrito aos pacientes com os seguintes requisitos: estar afebril por 24 horas; não estar em uso de drogas vasopressoras; não apresentar problemas na absorção e motilidade gastrintestinal; estar com prescrição de dieta oral e tolerar a alimentação; não estar em uso de antieméticos nas últimas 24 horas; e estar em uso de outros medicamentos por VO.^(^^9^^,^^16^^)^ Para esses pacientes, o farmacêutico realizava anotação no prontuário, orientando ao prescritor a substituição da via IV pela VO, por ser esta mais adequada ao quadro clínico do paciente.

Após as avaliações e as respectivas anotações em prontuário, o farmacêutico monitorava as prescrições dos pacientes, a fim de verificar a adesão do prescritor às recomendações por um período de 7 dias. A conduta do prescritor após a orientação do farmacêutico foi categorizada em “intervenção aceita”, quando o prescritor alterava a via de administração ou o diluente, conforme orientações do farmacêutico, ou “intervenção não aceita”, quando, após a orientação do farmacêutico, o prescritor mantinha a via de administração ou o diluente. Caso não houvesse adesão, o farmacêutico realizava nova avaliação da prescrição após esse período e registrava as intervenções em prontuário.

Os dados foram coletados por meio de pesquisa em prontuários em formulários específicos desenvolvidos pelo Serviço Farmacêutico Clínico Vertical. As informações geradas foram processadas e analisadas por meio dos softwares Epi Info™ versão 3.5.4, e STATA versão 12.0. As associações entre as variáveis foram testadas por meio dos testes χ^2^ de Pearson, exato de Fisher e o de McNemar, com nível de significância de 5%.

## RESULTADOS

Foram incluídos durante o período de estudo 978 prescrições de pacientes contendo omeprazol intravenoso. Destas, foram excluídas 10, pelo fato de o paciente ter recebido alta, e 198, devido ao prontuário do paciente não estar disponível no momento da avaliação pelo farmacêutico, totalizando 770 prescrições analisadas.

Foram avaliadas prescrições de 337 pacientes. Observou-se discreta predominância de pacientes do sexo feminino (Tabela 1). A média de idade dos pacientes foi de 55,12 anos (±18,01 anos), mais da metade dos pacientes tinham idade menor que 60 anos, e o aumento da idade mostrou-se fator de proteção (p=0,038) para a utilização inadequada da via IV. Em relação à especialidade do prescritor, predominou inadequação da via de administração do omeprazol dentre aqueles cujas especialidades eram cirúrgicas.

**Tabela 1:** Prevalência da inadequação da via de administração e diluente do omeprazol intravenoso

Variáveis		n=337	Via inadequada			Diluente inadequado		
		n (%)	n (%)	OR (IC95%)	Valor de p	n (%)	OR (IC95%)	Valor de p
Sexo								
	Masculino	153 (45,40)	40 (26,14)	0,92 (0,82-1,04)	0,189*	12 (7,84)	1,00	0,432*
	Feminino	184 (54,60)	37 (20,11)	1,00		19 (10,33)	0,97 (0,90-1,04)	
Idade, anos								
	Até 39	77 (22,85)	12 (15,58)	1,00	0,038*	7 (9,09)	0,99 (0,90-1,09)	0,955*
	40-59	105 (31,16)	20 (19,05)	0,95 (0,83-1,09)		9 (8,57)	1,00	
	Acima de 60	155 (45,99)	45 (29,03)	0,84 (0,73-0,96)		15 (9,68)	0,98 (0,91-1,06)	
Especialidade do prescritor								
	Médica	118 (35,01)	40 (33,90)	1,00	0,000*	13 (11,02)	0,96 (0,89-1,04)	0,397*
	Cirúrgica	219 (64,99)	37 (16,89)	1,25 (1,09-1,44)		18 (8,22)	1,00	

* teste χ^2^ de Pearson.

OR: *odds ratio*; IC95%: intervalo de confiança de 95%.

Em 13,12% (n=101) das prescrições avaliadas, a via de administração do omeprazol foi considerada inadequada para a condição clínica do paciente. Dentre as prescrições cuja via de administração foi considerada adequada, a restrição de dieta pela VO (514; 73,22%), problemas de motilidade (143; 20,37%) e problemas de absorção (45; 6,41%) foram os motivos mais frequentes. Vale ressaltar que, em alguns casos, os pacientes apresentaram mais de um motivo para justificar a adequação da utilização da via IV.

Em relação à adequabilidade do diluente prescrito, em 5,06% (n=39) das prescrições avaliadas, observou-se inadequação do tipo do diluente. Vale ressaltar que em 100,00% (n=39) dos casos de inadequação do tipo do diluente, observou-se que foi com água destilada.

De um total de 101 prescrições em que o farmacêutico avaliou e considerou a via de administração IV como inadequada, em 39,60% (n=40) das vezes a intervenção foi aceita pelo prescritor. Em relação às 29 prescrições em que o farmacêutico verificou o uso de diluente inadequado, 44,83% (n=13) das intervenções foram aceitas pelo prescritor. A intervenção farmacêutica para substituição do diluente do medicamento foi mais aceita pelos prescritores do que a intervenção para adequação da via de administração (p=0,000) (Tabela 2).

**Tabela 2:** Prevalência da aceitação das intervenções farmacêuticas quanto à terapia sequencial e à substituição do diluente do omeprazol intravenoso

Intervenções	Terapia sequencial	Substituição do diluente	Valor de p
Aceita	40 (39,60)	13 (44,83)	0,000*
Não aceita	61 (60,40)	16 (55,17)	
Total	101	29	

Resultados expressos como n (%).

* Teste McNemar.

## DISCUSSÃO

Os resultados deste estudo demonstram que as intervenções farmacêuticas podem colaborar para o uso correto dos medicamentos em ambiente hospitalar. Outros estudos que propuseram o monitoramento da adequabilidade da via de administração de medicamentos pelo farmacêutico também observaram a importância do acompanhamento contínuo na promoção do uso racional de medicamentos por via IV.^(^^17^^)^ Sabe-se que a presença do farmacêutico é uma estratégia de segurança,^(^^15^^)^ pois contribui com a promoção do uso correto de medicamentos no ambiente hospitalar.^(^^18^^)^

Embora a presença do farmacêutico seja, de fato, uma estratégia de segurança fortemente recomendada e aceita em outros países, não é uma realidade para grande parte do Brasil. O farmacêutico enfrenta dificuldades tanto de apoio da gestão, quanto pela aceitação, por parte da própria equipe multiprofissional.^(^^19^^)^ Essa realidade também foi observada no presente estudo, uma vez que as intervenções farmacêuticas que se relacionavam mais à avaliação clínica por parte do farmacêutico tiveram menor adesão por parte da equipe.

A aceitação das intervenções farmacêuticas no presente estudo, em geral, quando comparadas a outra instituição brasileira^(^^20^^)^ foi discrepante, uma vez que, nesse estudo, os autores observaram taxa de aceitação menor das intervenções. Tais achados podem ser parcialmente atribuídos ao fato de a comunicação das intervenções farmacêuticas ser registrada em prontuário físico, e pelo tempo estipulado para a reavaliação pelo farmacêutico. Esses fatores podem ter contribuído para a falta de adesão às intervenções. Além disso, a diferença do reconhecimento do papel do farmacêutico em ambas as instituições, e o potencial desse tipo de serviço contribuir com a segurança do processo de medicação e a prevenção de EAM^(^^15^^)^ também podem ter contribuído para esses resultados.

A baixa adesão às intervenções referentes à adequação da via de administração pode também ser devida ao fato de que, em ambiente hospitalar, a via IV é preferencialmente utilizada. Esta é uma via na qual o medicamento já apresenta resposta terapêutica imediata, bem como pela comodidade de o paciente já estar puncionado e, portanto, com uma via rápida disponível.^(^^6^^)^ Há que se ressaltar que embora de grande praticidade, a utilização inapropriada da via IV pode acarretar outros problemas que impactam na qualidade da assistência, como flebites e infecção de corrente sanguínea.^(^^8^^)^

Além disso, os erros oriundos do processo de medicação também contribuem para um aumento da morbimortalidade de pacientes hospitalizados. A reconstituição de antimicrobianos com cloreto de potássio tem acontecido em instituições hospitalares brasileiras^(^^7^^)^ e, embora raros de acontecerem, os desfechos são, em sua maioria, fatais. Práticas seguras para a utilização da via EV devem ser adotadas, estimuladas e amplamente difundidas nas instituições de saúde. Tal via de administração, além dos riscos apresentados, é mais dispendiosa, pois envolve custos diretos com diluentes, seringas, agulhas, dispositivos de infusão intravenosa, além dos custos indiretos com o tempo da equipe de enfermagem, que prepara as doses de medicamentos.^(^^8^^,^^9^^)^

Por outro lado, a aceitação das intervenções farmacêuticas referentes a aspectos farmacotécnicos da terapêutica, como o reconhecimento do equívoco relacionado ao diluente, pode ser mais compreensível por parte da equipe. Tal fato sugere que, para essas equipes, o papel do farmacêutico esteja mais relacionado à formulação dos medicamentos do que com a prática clínica, uma vez que o componente curricular do curso de farmácia representa uma barreira à sua atuação clínica no ponto de vista do médico.^(^^21^^)^

Ainda que a avaliação do diluente não demande, por parte do farmacêutico, conhecimentos essencialmente clínicos, no caso do omeprazol, as repercussões da utilização do diluente inadequado podem influenciar nos desfechos em saúde. Sabe-se que a estabilidade do omeprazol é pH-dependente, e sua reconstituição só deve ser feita com o diluente próprio, que acompanha o produto. Caso qualquer outro diluente seja utilizado na reconstituição do omeprazol, pode haver interferência na estabilidade da solução final e a eficácia do medicamento pode ficar prejudicada.^(^^22^^)^

As diferentes características dos profissionais pertencentes às especialidades médicas e cirúrgicas mostraram-se evidentes no presente estudo. A escolha adequada da via de administração do medicamento pressupõe uma relação médico-paciente mais estreita, que é comumente observada nas especialidades médicas.^(^^23^^)^ Por outro lado, a característica marcante dos cirurgiões, que é a praticidade e o pragmatismo,^(^^24^^)^ pode não favorecer o trabalho em equipe. Há que se promoverem estratégias para a sensibilização de membros dessa especialidade à adesão a estratégias relacionadas ao uso seguro de medicamentos.

Em relação a idade como um fator de proteção para a utilização inadequada da via IV, tal achado pode estar associado ao fato de que, comumente, a equipe médica tem maior cautela a prescrever para pacientes idosos.^(^^25^^)^ Aliado a isso, sempre que possível, a via IV deve ser evitada em pacientes idosos. Porém quando há necessidade de administrar medicamentos por via parenteral, deve-se avaliar a via subcutânea − técnica comumente conhecida como hipodermóclise, uma alternativa para a via IV.^(^^26^^)^ Esses achados podem ainda ser explicados por resultados do próprio estudo, uma vez que as especialidades médicas estiveram mais associadas aos cuidados de pacientes idosos no hospital em estudo.

Os pontos fracos do presente estudo consistem no fato de a instituição não possuir prontuário eletrônico, o que pode ter contribuído para a baixa adesão às intervenções farmacêuticas, uma vez que estas eram registradas em prontuário físico e só seriam lidas por parte da equipe médica na próxima visita. Outra limitação também observada foi a carga horária do farmacêutico para o desempenho das atividades, que era de 10 horas semanais. Vale ressaltar que, ainda sem recursos tecnológicos disponíveis e limitada carga horária, o farmacêutico foi capaz de avaliar expressivo número de prescrições e fazer as respectivas anotações em prontuário, além de registrar as intervenções. Esse modelo de serviço pode servir de subsídios para instituições que não contam com prontuário eletrônico implantar um serviço de farmácia clínica.

Como pontos fortes, destaca-se o caráter prospectivo da avaliação, que elimina os vieses característicos de pesquisas retrospectivas, além do fato de o serviço farmacêutico clínico vertical ter sido implantado em duas unidades de internação de uma só instituição. Perspectivas de estudos futuros nessa temática poderiam, além de avaliar a adequabilidade da via de administração dos IBP, checar as indicações de uso, uma vez que são poucos os estudos que têm avaliado o uso racional dessa classe de medicamentos em ambiente hospitalar.

Há que se instituir ainda programas de treinamento e sensibilização das equipes quanto ao uso racional dos inibidores da bomba de prótons. Embora esses medicamentos sejam amplamente utilizados na prática clínica, uma criteriosa avaliação do risco-benefício pode ser uma estratégia de prevenção de futuras complicações relacionadas ao seu uso prolongado.

Nesse contexto, o farmacêutico pode contribuir com a equipe de diversas maneiras: colaborando na elaboração de protocolos e diretrizes de utilização de medicamentos na instituição, na sensibilização e treinamento quanto ao uso racional dos medicamentos e participando das visitas ou na dispensação dos medicamentos.

## CONCLUSÃO

As intervenções farmacêuticas para a promoção do uso racional do omeprazol via endovenosa, embora parcialmente aceitas, puderam contribuir com a melhoria da segurança dos pacientes, uma vez que permitiram a utilização de uma via de administração mais segura. Houve diferenças em aceitabilidade das intervenções farmacêuticas relacionadas a utilização do diluente correto em detrimento daquelas relacionadas à troca da via de administração. A utilização inadequada da via intravenosa foi maior entre os cirurgiões em comparação aos clínicos, e o aumento da idade mostrou-se fator de proteção para a correta utilização da via intravenosa.
